# A Network Pharmacology Study Based on the Mechanism of Citri Reticulatae Pericarpium-Pinelliae Rhizoma in the Treatment of Gastric Cancer

**DOI:** 10.1155/2021/6667560

**Published:** 2021-04-16

**Authors:** Siyuan Song, Wenjie Huang, Xiaona Lu, Jiatong Liu, Jiayu Zhou, Ye Li, Peng Shu

**Affiliations:** ^1^Affiliated Hospital of Nanjing University of Chinese Medicine, Nanjing 210029, Jiangsu Province, China; ^2^Nanjing University of Chinese Medicine, Nanjing 210029, Jiangsu Province, China; ^3^Jiangsu Provincial Hospital of Chinese Medicine, Nanjing 210029, Jiangsu Province, China

## Abstract

**Objective:**

To explore the mechanism of action of Citri Reticulatae Pericarpium-Pinelliae Rhizoma (CRP-PR) in treating gastric cancer (GC) by using pharmacology network.

**Methods:**

Based on oral bioavailability and drug-likeness, the main active components of CRP-PR were screened using the Traditional Chinese Medicine Systems Pharmacology Database and Analysis Platform (TCMSP). DisGeNET Database was used to establish target databases for GC. Cytoscape software was used to construct a visual interactive network diagram of “Active Component-Target” and screen out the key targets. The STRING database was used to construct a protein interaction network. Gene Ontology (GO) and Kyoto Encyclopedia of Genes and Genomes (KEGG) pathway enrichment analysis were performed on the key targets. Additionally, TCGA and HPA databases were used for key target verification.

**Results:**

Thirty-seven active components of CRP-PR were screened. The results of network analysis showed that the main components include 8-octadecenoic acid, stigmasterol, ferulic acid, and naringenin of the CRP-PR herb pair. The key targets of the PPI network mainly involved GAPDH, MAPK3, JUN, STAT3, GSK3B, SIRT1, ERBB2, and SMAD2. GO enrichment analysis involves 540 biological processes, 118 cellular components, and 171 molecular functions. CRP-PR components were predicted to exert their therapeutic effect on the tumor signaling pathway, PI3K-Akt signaling pathway, MAPK signaling pathway, and estrogen signaling pathway. The validation of the key genes in the TCGA and HPA database showed that most of the key target verification results were consistent with this article.

**Conclusion:**

CRP-PR can treat GC by mediating PI3K-Akt signal pathway, MAPK signal pathway, and other biological processes such as tumor cell proliferation, apoptosis, and vascular regeneration, which embodies the synergistic effect of multi-components, multi-targets, and multi-channels, and provides the theoretical basis and research ideas for further study of CRP-PR in treating GC. 8-octadecenoic acid, stigmasterol, ferulic acid, and naringenin may be the material basis for the treatment of GC.

## 1. Introduction

Gastric cancer (GC), a primary epithelial-derived malignancy of the stomach, is the third most common cause of cancer death, second only to lung cancer and liver cancer [1]. GC is atypical with only epigastric discomfort in the initial stage. When the symptoms are obvious, the lesions usually have developed to the advanced stage where the methods of treatment are limited, including chemotherapy, immunotherapy, and targeted therapy [2]. Although progress has been improved in the diagnosis and treatment of GC, the prognosis of patients with GC is still poor, and the five-year survival rate is only 20%–30% [3].

It has been verified that traditional Chinese medicine (TCM) has the characteristics of “multiple components, multiple targets, and multiple pathways” in the treatment of various diseases [4]. Citri Reticulatae Pericarpium-Pinelliae Rhizoma (CRP-PR) herb pair, as the core components and classical herb pair of TCM compounds for the treatment of GC, is often used in combination, which can effectively improve the symptoms of GC and has been widely used in clinical practice [5–8].

For example, CRP-PR herb pair can treat coronary microcirculation disorders by protecting the function of coronary microcirculation endothelial cells and affecting the inflammatory response [9]. It can also promote lung cancer cell apoptosis by upregulating the expression of p53 and its downstream genes [10]. Besides, it can also be used to treat epilepsy [11], atherosclerosis [12], and other diseases.

PR is pungent in flavor and warm in nature, having the effect of drying dampness and dissolving phlegm, reducing adverse qi, stopping vomit, eliminating mass and resolving hard lump, detumescence, and relieving pain. The study has shown the PR can inhibit the invasion of cancer cells [13], Mao [14] has confirmed that PR extract can significantly reduce the invasion of BGC-823 cells through the Transwell invasion experiment. After treating tumor K562 cells with PR and PR extract, Guo found that it can interfere with DNA synthesis by prolonging the G0/*G*1 phase of tumor K562 cells and reduced the number of cells entering the DNA synthesis phase (S phase), leading to prolonged cell proliferation cycle, thereby inhibiting cell proliferation [15]. CRP is bitter, pungent, and warm in nature, having the effects of regulating qi, invigorating spleen, drying dampness, and resolving phlegm. Modern pharmacological studies have confirmed that nobiletin, an extract of CRP, has a wide range of pharmacological effects such as anti-tumor, anti-inflammation, and anti-oxidation [16, 17]. Nobiletin can inhibit the STAT3 pathway and then inhibit epithelial-mesenchymal transition (EMT), thereby reducing the invasion of SGC-7901 cells [18]. The main components of PR and CPR are *β*-sitosterol and tangerine peel, which have anti-inflammatory, anti-cancer, and antioxidant effects. Both of them can inhibit the superoxide anion produced in the xanthine oxidase/hypoxanthine system, and their ability to scavenge free radicals increases with the increase of the drug concentration within a certain concentration range. Both *β*-sitosterol and tangerines are good antioxidants, and they can scavenge free radicals and increase reactive oxygen species in cells, thereby acting on Akt and regulating cell proliferation and apoptosis [19–21].

As an emerging discipline that integrates bioinformatics, computer technology, pharmacology, and many other disciplines, network pharmacology can reveal the regulatory effects of various drugs on the body from the system level and played a vital role in promoting the modernization of TCM [22]. The development of TCM network pharmacology provides new research methods for the transformation of TCM from empirical medicine to an evidence-based medicine system, which will help explain the combination rules and network regulation effects of TCM, and can effectively discover new TCM active compounds [23]. Network pharmacology is the study of the biological basis of Chinese herbal medicine for cancer treatment; compounds in the same herbal medicine affect gene expression through the same or opposite pathways to regulate the occurrence and development of cancer [24]. Researching the core pathways of cancer development through network pharmacology will facilitate the discovery of new biomarkers and targets [25].

**Figure 1:**
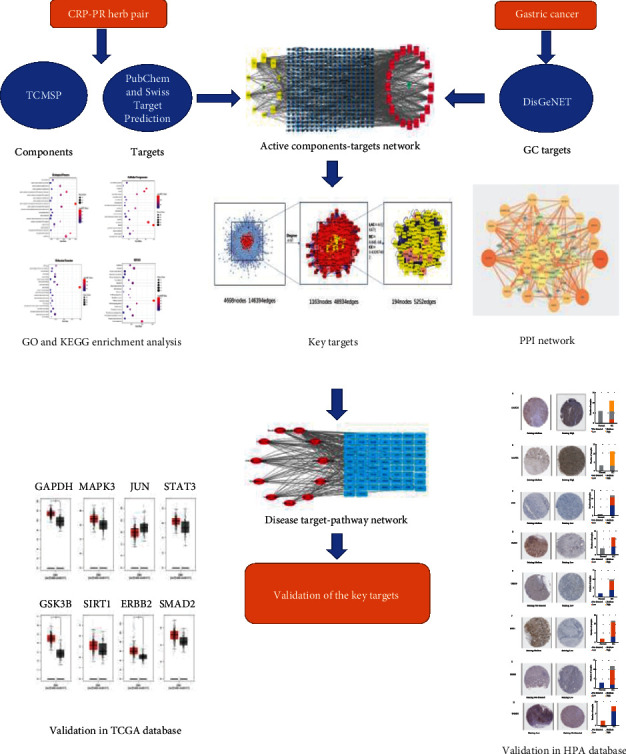
The protocol of our study procedures.

Network pharmacology elaborates the mechanism of multi-component, multi-target, and multi-pathway in treating diseases by herbs from micro to macro, which is consistent with the overall concept of traditional Chinese medicine [26]. CRP-PR herb pair is the main component of the JPYW. Our team has conducted an experimental study on the CRP-PR herb pair in the early stage. The results proved that CRP-PR herb pair was effective against BGC823/5-FU and BGC823 cells [27]. Both have a growth inhibitory effect, and the inhibitory effect increases with increasing concentration. However, there is no relevant modern pharmacological research to prove the specific targets and pathways of CRP-PR herb pair for the treatment of GC. Therefore, this paper mainly focuses on the research on the related targets and pathways of CRP-PR herb pair in the treatment of GC and provides a theoretical basis for later clinical trials. The protocol of our study procedures is shown in Figure 1.

## 2. Method

### 2.1. Active Components of CRP-PR

All active components of CRP-PR herb pair were retrieved from the Chinese Medicine System Pharmacology Database and Analysis Platform (TCMSP) [28] (http://lsp.nwu.edu.cn/tcmsp.php). In order to better screen the effective active compounds in the pair, the candidate active components of CRP-PR were obtained based on the criteria of drug-likeness (DL) [29] of ≥0.05 and oral bioavailability (OB) [30] of ≥30%. which are the key indicators of ADME (absorption, distribution, metabolism, and excretion) properties [31].

### 2.2. Prediction of Herb Targets for CRP-PR and Acquisition of GC Targets

The screened active components were transformed into the SMILES structure by the PubChem database (https://pubchem.ncbi.nlm.nih.gov/) and then imported into the Swiss Target Prediction website (http://www.swisstargetprediction.ch/) to predict all potential targets of CRP-PR. In this study, the probability TOP50 of each SMILES structure prediction target was selected as the criterion. The keywords “gastric cancer”, “gastric carcinoma”, “stomach cancer”, and “stomach carcinoma” were searched in DisGeNET (https://www.disgenet.org/home/) database to obtain GC-related targets.

### 2.3. Construction of “Active Component-Target” Network

The potential action targets of CRP-PR were obtained through the Swiss Target Prediction website. Gene targets and corresponding active components were input into Cytoscape software to construct an “Active Component-Target” network.

### 2.4. Key Targets Screening

The target set of components of CRP-PR and the target set of GC were imported into Cytoscape software for intersection, taking the topological feature value “Degree” of network nodes as an index, the nodes with degree value more than twice their median were selected as key candidate targets, and then three topological feature values “medium centrality (BC),” “close centrality (CC),” and “local edge connectivity (LAC)” of network nodes were calculated based on CytoNCA plug-in to extract these candidate node network relations; the key targets of CRP-PR herb pair for treating GC were screened by selecting the above three nodes whose eigenvalues were greater than their corresponding median.

### 2.5. Protein-Protein Interaction (PPI) Network Construction

The key targets of CRP-PR for GC treatment were imported into the STRING database (https://string-db.org/cgi/input.pl), and the PPI network was constructed.

### 2.6. Enrichment Analysis of GO and KEGG

The targets of diseases treated by herbs were imported into the David database (https://david.ncifcrf.gov/), and the species was defined as *Homo sapiens* for GO and KEGG enrichment analysis. GO enrichment analysis consists of three parts: biological process (BP), cellular component (CC), and molecular function (MF). *P* < 0.05 and error detection rate (FDR) < 0.05 were used for GO enrichment analysis; *P* < 0.01 was used for KEGG enrichment analysis.

### 2.7. Construction of “Disease Target-Pathway” Network

The pathways such as “hepatitis B″ and “prostate cancer” that were not related to GC in the first 20 KEGG enrichment analyses were excluded, and the network diagram of “Disease Target-Pathway” was constructed.

### 2.8. Validation of Key Genes in TCGA and HPA Database

The 8 key genes were input into the online tool of GEPIA (http://gepia.cancer-pku.cn/index.html) to verify its expression in TCGA-STAD. And then the protein expression and distribution of 8 key genes were investigated in HPA database (https://www.proteinatlas.org/).

## 3. Results

### 3.1. Active Components of CRP-PR

A total of 37 components of CRP-PR were retrieved from the TCMSP database, including 11 CRP and 20 PR. Basic information of the 37 active components is shown in Table 1.

### 3.2. Prediction of CRP-PR Targets and Acquisition of GC Targets

A total of 300 potential targets of CRP active components and 919 potential targets of PR active components were obtained from the database prediction and screening. The average value of score gad (gene-disease association score) was calculated, and a total of 634 GC targets with a score gad >0.053551136 were selected.

### 3.3. Construction of “Active Component-Target” Network

**Figure 2:**
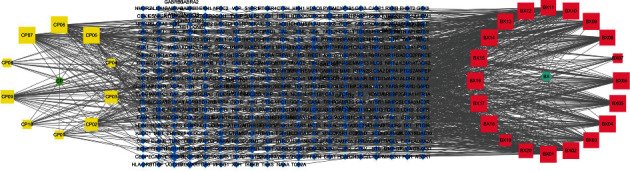
Network diagram of CRP-PR “Active Component-Target.”

The components and corresponding targets were imported into Cytoscape software to obtain the network diagram of “Active Component-Target,” shown in Figure 2.

### 3.4. Key Targets Screening

**Figure 3:**
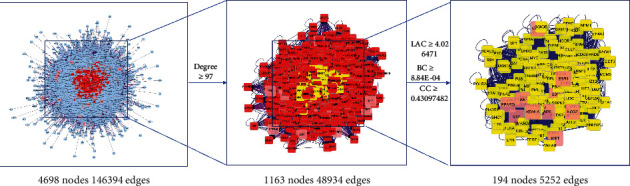
Key targets of CRP-PR in the treatment of GC.

The intersection network included 4698 nodes and 146394 edges. The median degree value was 48.5, so the nodes with “Degree ≥97 (2 times the median value)” were selected as the candidate targets after topology analysis. The key targets were further screened with “BC ≥ 8.84E-04, CC ≥ 0.430975, and LAC ≥ 4.026471”. Finally, a total of 303 key targets of CRP-PR for the treatment of GC were obtained, as shown in Figure 3.

### 3.5. Protein-Protein Interaction Network Construction and Analysis

**Figure 4:**
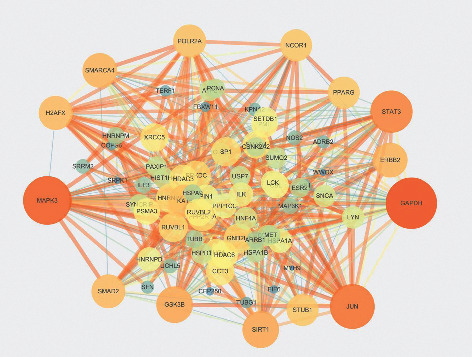
Protein-protein interaction network.

The PPI network diagram was constructed with Cytoscape software. Node size and color were set to reflect the degree of freedom of the node (degree), and the thickness of the edge represented the combined score (Figure 4). GAPDH, MAPK3, JUN, STAT3, GSK3B, SIRT1, ERBB2, and SMAD2 were at the core sites.

### 3.6. Enrichment Analysis of GO and KEGG

**Figure 5:**
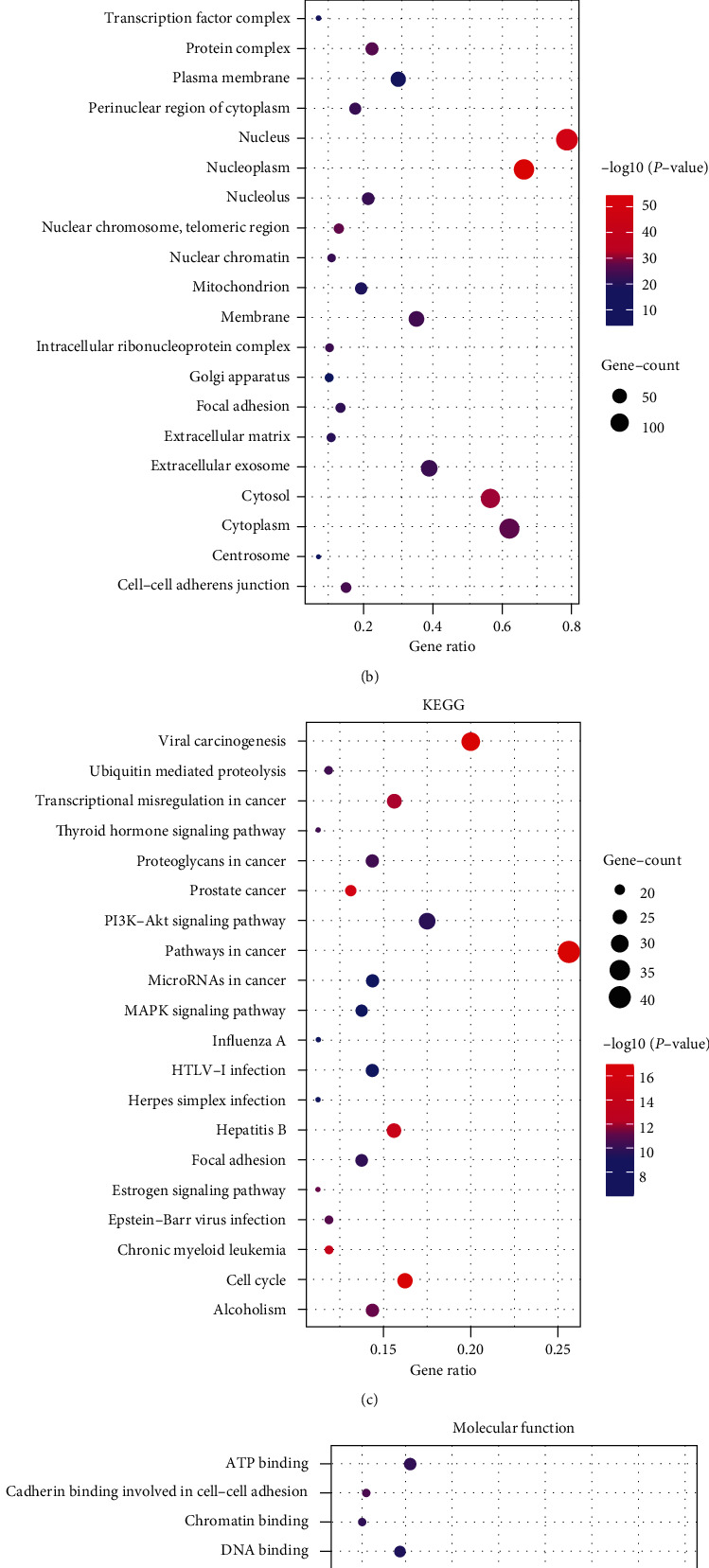
Bubble diagram for GO and KEGG enrichment analysis. (a) Biological process. (b) Cellular components. (c) Molecular function. (d) KEGG.

The biological function enrichment analysis of GO and KEGG was performed on 303 targets involved in the treatment of GC by David software. The results showed that the predicted target genes of CRP-PR against active components were mainly enriched in 540 biological processes, 118 cellular components, and 171 molecular functions, biological processes involving mainly the positive regulation of transcription from RNA polymerase II promoter and negative regulation of transcription from RNA polymerase II promoter transcription. Cell components mainly include the nucleus, cytoplasm, and cytosol. Molecular functions mainly include protein binding, DNA binding, and ATP binding. Through KEGG enrichment analysis, 103 significantly enriched pathways were identified, which were mainly involved in the tumor signaling pathway, PI3K-Akt signaling pathway, cell cycle, and MAPK signaling pathway. The results of GO and KEGG analysis were ranked as TOP20 from high to low in count value. TOP20 were plotted into bubble charts using *R* language, where the bubble size represents the number of enriched genes and the bubble color difference represents the significant magnitude of target gene enrichment, as shown in Figure 5.

The bubble size represents the number of enriched genes, and the bubble color difference represents the significant magnitude of target gene enrichment.

### 3.7. Construction of “Disease Target-Pathway” Network

**Figure 6:**
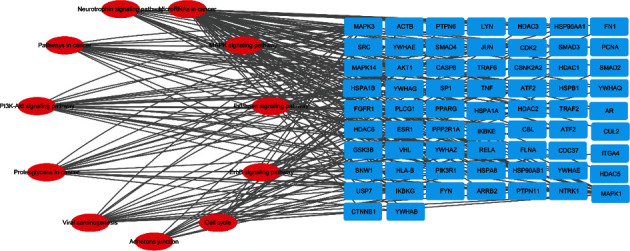
Construction of “disease target-pathway” network.

The GC-related pathways and target information in the first 20 KEGG enrichment were input into Cytoscape, and the “Disease Target-Pathway” network diagram was drawn as shown in Figure 6.

### 3.8. Validation of the Key Genes in TCGA and HPA Database

**Figure 7:**
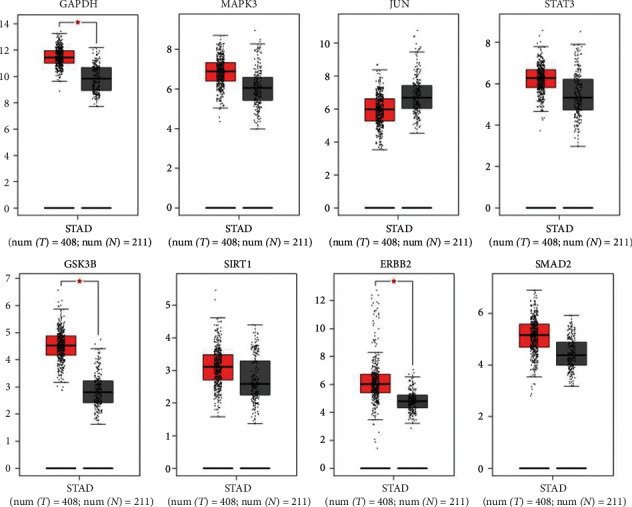
Expression of the key genes in TCGA database. The box plots show the genes expression in GEPIA. Red represents tumor, and gray represents normal.

**Figure 8:**
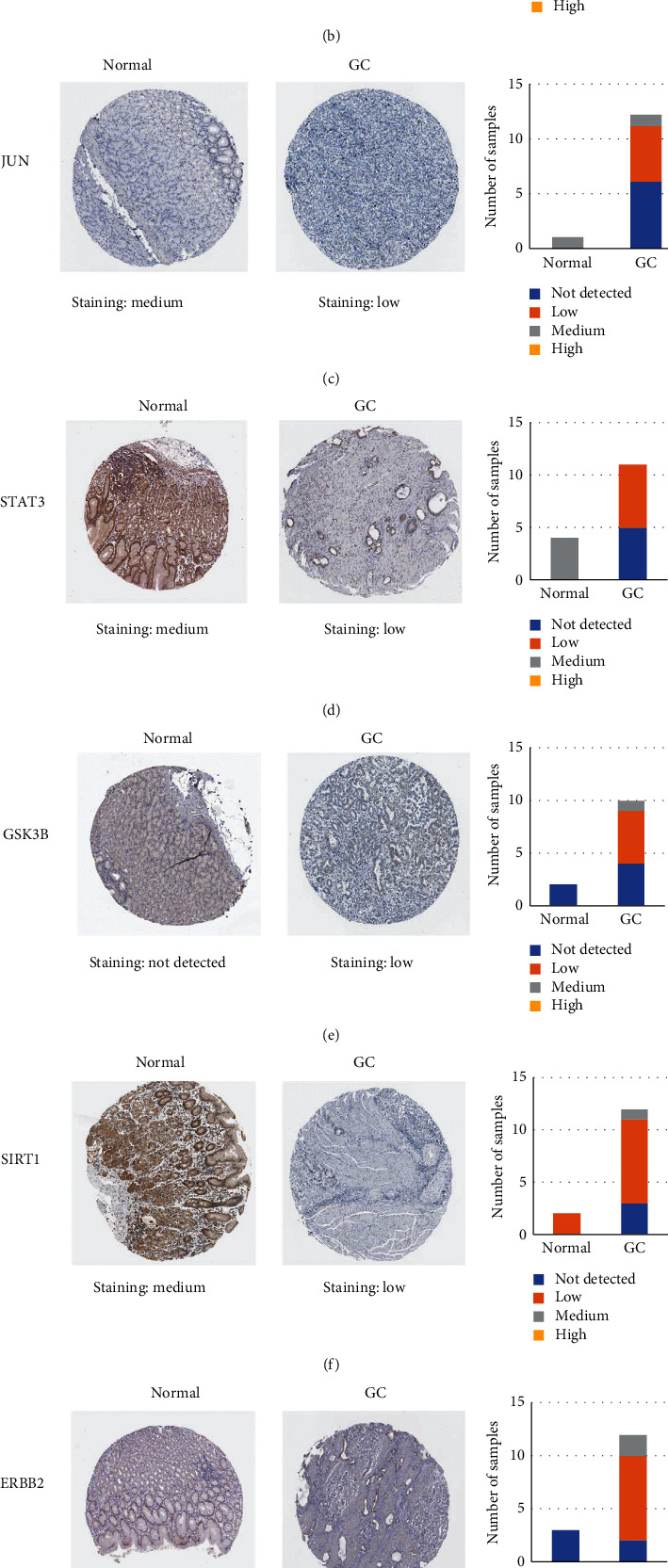
Validation of the key genes in the HPA. Representative immunohistochemistry images of (a) GAPDH, (b) MAPK3, (c) JUN, (d) STAT3, (e) GSK3B, (f) SIRT1, (g) ERBB2, and (h) SMAD2 in GC and noncancerous stomach tissues derived from the HPA database. The staining strengths were annotated as not detected, low, medium, and high. The bar plots indicate the number of samples with different staining strengths.

GEPIA database was used to view GAPDH, MAPK3, JUN, STAT3, GSK3B, SIRT1, ERBB2, and SMAD2 in the STAD samples in the TCGA database. The results showed that GAPDH, GSK3B, and ERBB2 were highly expressed in GC tissues (Figure 7). The HPA online tool was used to analyze the protein expression of 8 key genes (Figure 8). The results showed that the GAPDH and MAPK3 protein genes were medium expressed in normal gastric tissues and were highly expressed in GC tissues. The JUN, STAT3, and SIRT1 protein genes were medium expressed in normal gastric tissues and lowly expressed in GC tissues. The GSK3B and ERBB2 protein genes were not detected in normal gastric tissues, but lowly expressed in GC tissues. The SMAD2 protein gene was lowly expressed in normal gastric tissues, but not detected in GC tissues.

## 4. Discussion

GC can be classified into such categories as “epigastric pain,” “accumulation,” and “abdominal mass” in TCM. The masses are usually formed due to the retention of turbid phlegm, blood stasis, and heat toxin in the stomach for a long time. The CRP-PR are the core composition and classic pair of Chinese medicinal compounds for the treatment of GC, which have been widely used in clinical practice. CRP-PR herb pair are the main ingredients of the prescription for Jianpi Yangwei decoction (JPYW). Our team has done many experiments in vivo and in vitro to verify the therapeutic effect of JPYW prescription on GC in the early stage [32–36]. The JPYW was created by the first nationally renowned Chinese medicine doctor Shenlin Liu based on the theory of “spleen deficiency, blood stasis, and toxin.” 489 cases were included. The research results confirmed that the TCM treatment combined with chemotherapy with JPYW as the main prescription had the effect of reducing the risk of recurrence and metastasis in patients with GC stage II/III after surgery. Compared with the chemotherapy alone group, the risk of recurrence and metastasis was reduced by 32.8% (*P*=0.0042). Especially for patients with stage III GC, the percentage of reduction in the risk of recurrence and metastasis increased to 34.7% (*P*=0.0072) [37]. Tang [27] used MTT, Hoechst fluorescent staining, Tunel, and other detection methods and found that JPYW induced apoptosis of BGC823 cells and BGC823/5-Fu cells and downregulated the expression of resistance proteins MDR1, MRP1, and ABCG2 in vivo and in vitro. Its effect in vitro was related to PI3K/Akt signaling pathway. CRP-PR had an inhibitory effect on the growth of BGC823 cells, and the inhibition rate increased with the increase in concentration [38].

The CRP-PR herb pair played a one-to-many role in regulating and controlling GC in the study. Analysis of the “Active Components-Targets” revealed that 8-octadecenoic acid, stigmasterol, ferulic acid, and naringenin might be the main components of the CRP-PR herb pair for the treatment of GC. 8-octadecenoic acid with a mass fraction ≥0.2 g kg^−1^ was injected into severe combined immunodeficiency (SCID) mice. It was observed that 8-octadecenoic acid had a significant inhibitory effect on the growth of GC transplanted tumors [39]. Stigmasterol can induce apoptosis of SGC-7901 cells by promoting the expression of caspase-3. The growth of SGC-7901 cells in the phytosterol treatment group with a concentration of 2.8 *μ*g/mL–44.8 *μ*g/mL was inhibited to varying degrees. The higher the dose of phytosterol, the more obvious the inhibitory effect [40]. Ferulic acid with concentrations of 5, 7.5, and 10 mg/mL interfered with MGC-803 cells in vitro, and the results showed that ferulic acid can make MGC-803 cells Caspase-3, Caspase-9, Bax mRNA, and protein expression upregulated differently. The mRNA and protein expressions of Bcl-2 and Xiap were downregulated to varying degrees, indicating that ferulic acid may decrease the mitochondrial membrane potential through changes in the expression of Bcl-2 and Bax proteins, and activated Caspase-9 and Caspase-3 to cause cell apoptosis. Therefore, ferulic acid induced apoptosis of MGC-803 cells and effect through the mitochondrial pathway [41]. Naringenin is the largest polyphenol compound with anti-inflammation, immune regulation, and anti-cancer effects [42], which have a certain inhibitory effect on SGC-7901 cells [43]. 200 and 400 *μ*mol/L naringenin can significantly reduce the adhesion, invasion, and migration ability of GC cells (*P* < 0.05), while 100 *μ*mol/L naringenin can only reduce the migration ability of GC cells (*P* < 0.05), but had no obvious effect on adhesion and invasion ability [44].

GAPDH, MAPK3, JUN, STAT3, GSK3B, SIRT1, ERBB2, and SMAD2, which are at the key of the PPI network, are important targets of CRP-PR herb pair for the treatment of GC. The validation of the key genes in the TCGA and HPA database showed that GAPDH, GSK3B, and ERBB2 were highly expressed in GC tissues. GAPDH and MAPK3 protein genes were medium expressed in normal gastric tissues and were highly expressed in GC tissues. The JUN, STAT3, and SIRT1 protein genes were medium expressed in normal gastric tissues and lowly expressed in GC tissues. The GSK3B and ERBB2 protein genes were not detected in normal gastric tissues, but lowly expressed in GC tissues. The SMAD2 protein gene was lowly expressed in normal gastric tissues, but not detected in GC tissues. Most of the above verification results were consistent with this article. It has been demonstrated that extracellular GAPDH (glyceraldehyde-3-phosphate dehydrogenase) or its N-terminal domain can inhibit the growth of GC cells [45], and the negative regulation of tumor growth with GAPDH may be a new anti-cancer strategy [46]. Yamaji [47] reported that GAPDH was secreted by some cancer cells and could inhibit cell proliferation. Yang [48] showed that the expression level of GAPDH in GC was higher than that in normal tissues. MAPK3 is a member of the MAPK family. MAPK is an important signal transmitter in cells that can participate in a variety of biological processes such as cell proliferation, differentiation, and immune defense by phosphorylating nuclear transcription factors and related enzymes [49]. The abnormal expression of MAPK3 was related to the invasion, metastasis, and drug resistance of a variety of tumor cells. Kim [50] found that MAPK3 expression is an independent prognostic index for patients after gastrectomy. JUN is a protein family that constitutes transcription factor AP-1 (activator protein-1), including c-JUN, v-JUN, JUN-B, and Jun-D. Studies have shown that AP-1 was involved in the regulation of various cellular processes, such as cell proliferation, differentiation, and apoptosis through the transcription of various growth factors, and cytokines [51]. Transcription factor JUN is a risk gene for GC, and it can promote the occurrence and development of GC by participating in the regulation of the MAPK signaling pathway [52]. STAT3 is a signal transduction and transcription activation factor 3. The significant increase of STAT3 is related to the occurrence and development of GC. STATA3 is valuable for the early diagnosis of GC. Duan [53] found that the differential expression of STAT3 in GC was closely related to pathological features such as cancer tissue infiltration and lymph node metastasis, and some studies have suggested that this target was related to the treatment and prognosis of GC [54]. STAT3 acts as a carcinogen in GC, which can enhance the metastatic potential of tumor cells and promote the development and progression of tumors [55]. GSK3B (glycogen synthase-3) is a serine/threonine kinase that widely exists in mammalian eukaryotic cells. GSK3B can act on many signal protein structural proteins and transcription factors to regulate cell differentiation, proliferation, and apoptosis. GSK3B participates in the regulation of apoptosis by affecting the glucose concentration in the blood and the ratio of Bax/HKII, which in turn affects mitochondrial permeability and the release of cytochrome C. Lukas [56] found that the prognosis of GC was closely related to the expression of GSK3B. The prognosis of patients with high expression of GSK3B in GC was better than that of patients with low expression of GSK3B. SIRT1 (silent information regulator (1) is a protein deacetylase that is related to the proliferation and apoptosis of tumor cells [57]. SIRT1 can accelerate the process of GC and aggravate the growth of tumors, making GC cells vulnerable to biological behaviors such as invasion and metastasis along with the rapid progress of the disease [58]. ERBB2 (tyrosine kinase receptor (2)) is one of the members of the epidermal growth factor receptor (EGFR) family. ERBB receptor family members activate receptor cytoplasmic tyrosine kinase domains by forming homologous or heterologous dimers. Their abnormal activation can recruit downstream signaling proteins and finally activate downstream signaling pathways such as Ras/Raf/MAPK, ERK1/ERK2, and JNK, thus promoting cell proliferation and angiogenesis and inhibiting cell-initiated apoptosis regulation [59]. Among them, the core position in the signaling network of the whole ERBB receptor family is ERBB2, which does not directly bind to any ERBB ligand, but it is in a conformation similar to the ligand activation state, which facilitates the formation of dimers with the rest of the ERBB receptors, and is the preferred target for dimerization of the rest of the receptors [60]. The abnormal expression of ERBB2 is closely related to the occurrence and development of a variety of malignant tumors, including GC [61]. SMAD2 is one of the members of the SMAD family of proteins. The SMAD protein family is a very important mediator in the intracellular signal transduction process of transforming the growth factor-1 (TGF-1) superfamily, which mainly mediates the TGF-*β*1 signaling pathway. The TGF-*β*1/SMAD pathway is involved in many biological processes [62] such as embryonic development, tumor occurrence, and development. The research by Liu [63] has revealed that SMAD protein is highly expressed in GC tissues, suggesting that SMAD gene expression is involved in GC transformation and plays a role in the occurrence of GC.

The KEGG pathway enrichment analysis excluded pathways unrelated to GC and revealed that the pathogenesis of gastric cancer was related to tumor signaling pathway, PI3K-Akt signaling pathway, cell cycle, MAPK signaling pathway, cancer transcription imbalance, and estrogen signaling pathway. In tumor cells, PI3K-Akt is one of the main signaling pathways that regulate proliferation, invasion, and migration [64]. PI3K/Akt signaling pathway plays an important role in the occurrence and development of GC. It has been proved that the PI3K-Akt signaling pathway can promote the proliferation and inhibit apoptosis of GC cells, which is closely related to the invasion and metastasis of gastric cancer cells [65]. MAPK pathway is one of the crucial signaling pathways involved in the regulation of proliferation, migration, and angiogenesis [66], which was involved in the regulation of the migration of gastric malignant tumors and GC cells [67]. Estrogen receptor −*α*36 (ER−*α*36)-mediated rapid estrogen signaling pathway plays an important role in the occurrence and development of GC. Estrogen can stimulate the growth of GC cells through ER−*α*36 signaling pathway. ER−*α*36 is highly expressed in GC tissues, and the expression of ER−*α*36 in intestinal GC is higher than that in diffuse GC [68].

## 5. Conclusion

The action mechanism of CRP-PR herb pair in the treatment of GC was studied by network pharmacology method of active component screening, network construction, and pathway analysis in this study. Then, we validated the key genes in TCGA and HPA database; most of the above verification results were consistent with this article. By comparing the research results with known experimental results, it was concluded that CRP-PR herb pair treat diseases by mediating the biological processes such as tumor signaling pathway, PI3K-Akt signaling pathway, MAPK, and others to regulate the proliferation, apoptosis, metastasis, and vascular regeneration of GC cells, reflecting the synergistic effect of Chinese medicine from multiple components, multiple targets, and multiple pathways, which will lay a solid foundation for further elucidation of its action mechanism. The deficiency is due to the prevalence of COVID-19, experimental conditions, time, and other constraints; we are unable to carry out relevant experimental verification in a short time. We will definitely conduct in-depth experimental research on the key targets and pathways of this article when conditions permit at a later period.

## Figures and Tables

**Table 1:** Information of the active components of CRP-PR.

Herb	Number	Mol ID	Component	OB	DL
CRP	CP01	MOL000125	(-)-Alpha-pinene	46.25	0.05
	CP02	MOL002029	()-Cuparene	38.26	0.07
	CP03	MOL002095	DEP	52.19	0.07
	CP04	MOL003538	()-Ledene	51.84	0.1
	CP05	MOL000359	Sitosterol	36.91	0.75
	CP06	MOL004328	Naringenin	59.29	0.21
	CP07	MOL005100	5,7-Dihydroxy-2-(3-hydroxy-4-methoxyphenyl) chroman-4-one	47.74	0.27
	CP08	MOL000057	DIBP	49.63	0.13
	CP09	MOL005815	Citromitin	86.9	0.51
	CP10	MOL005816	Alpha-sinensal	57.79	0.06
	CP11	MOL005828	Nobiletin	61.67	0.52

PR	BX01	MOL000131	EIC	41.9	0.14
	BX02	MOL001739	Zoomaric acid	35.78	0.1
	BX03	MOL001755	24-Ethylcholest-4-en-3-one	36.08	0.76
	BX04	MOL001818	Methyl palmitelaidate	34.61	0.12
	BX05	MOL002495	6-Shogaol	31	0.14
	BX06	MOL002670	Cavidine	35.64	0.81
	BX07	MOL002714	Baicalein	33.52	0.21
	BX08	MOL002776	Baicalin	40.12	0.75
	BX09	MOL000358	Beta-sitosterol	36.91	0.75
	BX10	MOL000389	FERULIC ACID (CIS)	54.97	0.06
	BX11	MOL000432	Linolenic acid	45.01	0.15
	BX12	MOL000449	Stigmasterol	43.83	0.76
	BX13	MOL005030	Gondoic acid	30.7	0.2
	BX14	MOL000519	Coniferin	31.11	0.32
	BX15	MOL000675	Oleic acid	33.13	0.14
	BX16	MOL006936	10,13-Eicosadienoic	39.99	0.2
	BX17	MOL006937	12,13-Epoxy-9-hydroxynonadeca-7,10-dienoic acid	42.15	0.24
	BX18	MOL006944	8-Octadecenoic acid	33.13	0.14
	BX19	MOL006951	Pedatisectine a	64.09	0.16
	BX20	MOL006952	Pedatisectine f	53.81	0.06
	BX21	MOL006956	cyclo-(leu-tyr)	111.16	0.15
	BX22	MOL006957	(3S, 6S)-3-(benzyl)-6-(4-hydroxybenzyl) piperazine-2,5-quinone	46.89	0.27
	BX23	MOL006958	cyclo-(val-tyr)	122.79	0.14
	BX24	MOL003578	Cycloartenol	38.69	0.78
	BX25	MOL006962	2Z-hexadecenoic acid	34.02	0.1
	BX26	MOL006967	beta-D-Ribofuranoside, xanthine-9	44.72	0.21

## Data Availability

The data used to support the findings of this study are included within the article.

## Abbreviations

GC: Gastric cancer
TCMSP: Traditional Chinese Medicine Systems Pharmacology Database and Analysis Platform
TCM: Traditional Chinese medicine
OB: Oral bioavailability
DL: Drug-likeness
BC: Medium centrality
CC: Close centrality
LAC: Local edge connectivity
GO: Gene ontology
BP: Biological process
CC: Cellular component
MF: Molecular function
FDR: Error detection rate
KEGG: Kyoto Encyclopedia of Genes and Genomes
PPI: Protein-protein interaction
EMT: Epithelial-mesenchymal transition
JPYW: Jianpi Yangwei decoction.
